# Assessment of Prevalence and Cost of Care Cascades After Routine Testing During the Medicare Annual Wellness Visit

**DOI:** 10.1001/jamanetworkopen.2020.29891

**Published:** 2020-12-11

**Authors:** Ishani Ganguli, Claire Lupo, Alexander J. Mainor, Qianfei Wang, E. John Orav, Meredith B. Rosenthal, Thomas D. Sequist, Carrie H. Colla

**Affiliations:** 1 Harvard Medical School, Boston, Massachusetts; 2 Division of General Internal Medicine and Primary Care, Brigham and Women’s Hospital, Boston, Massachusetts; 3 The Dartmouth Institute for Health Policy and Clinical Practice, Geisel School of Medicine at Dartmouth, Lebanon, New Hampshire; 4 Department of Biostatistics, Harvard T.H. Chan School of Public Health, Boston, Massachusetts; 5 Department of Health Care Policy and Management, Harvard T.H. Chan School of Public Health, Boston, Massachusetts; 6 Mass General Brigham, Boston, Massachusetts; 7 Norris Cotton Cancer Center, Dartmouth-Hitchcock Medical Center, Lebanon, New Hampshire

## Abstract

**Importance:**

For healthy adults, routine testing during annual check-ups is considered low value and may trigger cascades of medical services of unclear benefit. It is unknown how often routine tests are performed during Medicare annual wellness visits (AWVs) or whether they are associated with cascades of care.

**Objective:**

To estimate the prevalence of routine electrocardiograms (ECGs), urinalyses, and thyrotropin tests and of cascades (further tests, procedures, visits, hospitalizations, and new diagnoses) that might follow among healthy adults receiving AWVs.

**Design, Setting, and Participants:**

Observational cohort study using fee-for-service Medicare claims data from beneficiaries aged 66 years and older who were continuously enrolled in fee-for-service Medicare between January 1, 2013, and March 31, 2015; received an AWV in 2014; had no test-relevant prior conditions; did not receive 1 of the 3 tests in the 6 months before the AWV; and had no test-relevant symptoms or conditions in the AWV testing period. Data were analyzed from February 13, 2019, to June 8, 2020.

**Exposure:**

Receipt of a given test within 1 week before or after the AWV.

**Main Outcomes and Measures:**

Prevalence of routine tests during AWVs and cascade-attributable event rates and associated spending in the 90 days following the AWV test period. Patient, clinician, and area-level characteristics associated with receiving routine tests were also assessed.

**Results:**

Among 75 275 AWV recipients (mean [SD] age, 72.6 [6.1] years; 48 107 [63.9%] women), 18.6% (14 017) received at least 1 low-value test including an ECG (7.2% [5421]), urinalysis (10.0% [7515]), or thyrotropin test (8.7% [6534]). Patients were more likely to receive a low-value test if they were younger (adjusted odds ratio [aOR], 1.69 for ages 66-74 years vs ages ≥85 years [95% CI, 1.53-1.86]), White (aOR, 1.32 compared with Black [95% CI, 1.16-1.49]), lived in urban areas (aOR, 1.29 vs rural [95% CI, 1.15-1.46]), and lived in high-income areas (aOR, 1.26 for >400% of the federal poverty level vs <200% of the federal poverty level [95% CI, 1.16-1.37]). A total of 6.1 (95% CI, 4.8-7.5) cascade-attributable events per 100 beneficiaries occurred in the 90 days following routine ECGs and 5.4 (95% CI, 4.2-6.5) following urinalyses, with cascade-attributable cost per beneficiary of $9.62 (95% CI, $6.43-$12.80) and $7.46 (95% CI, $5.11-$9.81), respectively. No cascade-attributable events or costs were found to be associated with thyrotropin tests.

**Conclusions and Relevance:**

In this study, 19% of healthy Medicare beneficiaries received routine low-value ECGs, urinalyses, or thyrotropin tests during their AWVs, more often those who were younger, White, and lived in urban, high-income areas. ECGs and urinalyses were associated with cascades of modest but notable cost.

## Key Points

**Question:**

What are the prevalence of low-value testing during Medicare annual wellness visits and of cascades of medical services and new diagnoses that might follow?

**Findings:**

In this cohort study of 75 275 fee-for-service Medicare beneficiaries aged 66 and older who received an annual wellness visit, 19% of beneficiaries received at least 1 routine electrocardiogram, urinalysis, or thyrotropin test during their visit, more often those who were younger and lived in urban, high-income areas. An estimated 6 cascade-attributable events per 100 electrocardiogram recipients and 5 events per 100 urinalysis recipients occurred in the subsequent 90 days.

**Meaning:**

In this study, a minority of healthy Medicare beneficiaries received routine tests during their annual wellness visits, and low-value electrocardiograms and urinalyses were associated with cascades of care.

## Teaser

This cohort study uses US Medicare claims data to estimate the prevalence of routine electrocardiogram, urinalyses, and thyrotropin tests performed during annual wellness visits and assesses the prevalence and cost of laboratory tests, imaging tests, procedures, visits, hospitalizations, and new diagnoses that may follow.

## Introduction

For healthy adults, routine testing during annual check-ups is considered to be of low value.^1,2,3,4,5^ Organizations such as the United States Preventive Services Task Force and the Choosing Wisely campaign^4^ advise against routine electrocardiograms (ECGs),^6^ urinalyses,^7^ and thyrotropin tests^8^ (eAppendix 1 in the Supplement). While US studies from the 1990s and 2000s suggest that these tests were commonly performed^9,10^ and favored by physicians,^11^ we do not have more recent estimates. In addition, we do not know how often these tests are performed during the Medicare yearly check-up, known as the *annual wellness visit* (AWV) and introduced in 2011 through the Affordable Care Act.^12^

Routine medical tests during these visits may also trigger cascades of further medical services that are of uncertain value and may cause substantial harms to patients and clinicians.^1,13,14,15,16,17^ Understanding the prevalence of these tests and their potential cascades would allow payers, policy makers, and clinicians to prioritize and target efforts to mitigate low-value care and its consequences. We used national Medicare claims data to estimate the prevalence of routine ECG, urinalysis, and thyrotropin testing performed during AWVs, to identify patient, area-level, and visit characteristics associated with receiving these tests, and to measure the prevalence and cost of cascade-attributable laboratory tests, imaging tests, procedures, visits, hospitalizations, and new diagnoses that may follow.

## Methods

### Data Source

We analyzed a 20% random sample of fee-for-service Medicare beneficiary parts A and B claims data from January 1, 2013 to March 31, 2015. Data were analyzed from February 13, 2019, to June 8, 2020. This cohort study was waived for review by the institutional review boards at Dartmouth College and the Harvard T.H. Chan School of Public Health owing to use of deidentified data. This study followed the Strengthening the Reporting of Observational Studies in Epidemiology (STROBE) reporting guideline for cohort studies.

### Study Cohort

We identified beneficiaries who were age 66 years or older (to capture age-based enrollees eligible for an AWV after 1 year of enrollment) as of January 1, 2014; resided in the 50 US states; and were continuously enrolled in fee-for-service Medicare between January 1, 2013, and March 31, 2015. Among this group, we examined those who had an AWV (Healthcare Common Procedure Coding System codes G0438, G0439) between January 1 and December 31, 2014. To create a cohort of healthy asymptomatic patients without appropriate indications for any of the 3 tests of interest, we first excluded beneficiaries with a relevant chronic condition (eg, hypothyroidism) in the 365 days preceding their AWV. We further excluded patients with a symptom, acute condition, or 1 of the aforementioned chronic conditions within 7 days before or after their AWV (based on a diagnosis code billed in that period) that might suggest a diagnostic or other clinical indication for a test (eg, hematuria for urinalysis). We chose this time period to account for the common practice, which we confirmed empirically, of patients being tested in anticipation of or shortly following their visit. We further excluded patients who received any index test within 6 months before this testing period based on the assumption that for these patients, a repeat of this index test during the AWV would not constitute a routine test. Details, study timeline, and study cohort flow diagram are available in eFigure 1, eFigure 2, and eTable 1 in the Supplement.

### Measures

#### Index Events

Using prior literature and clinical judgment,^9^ we identified 3 tests that might be performed as routine tests during wellness visits and lead to potential cascades: ECG, urinalysis, and thyrotropin test (eAppendix 2, eTable 2 in the Supplement). We examined incidence of these index tests during the AWV test period (ie, from 7 days before to 7 days after the AWV).

#### Cascade Events

We defined cascade events as follow-up services and new diagnoses that would plausibly follow from the index event and could be captured reliably in claims data. Using prior literature, guidelines,^6,7,8,14,18^ and clinical knowledge, 2 internal medicine physicians/health services researchers (I.G. and T.D.S.) defined and reached consensus on cascade events that might arise from each initial test. We categorized services as laboratory tests, imaging tests (including noninvasive cardiac studies), procedures, visits, and hospitalizations. If a service could be classified into more than 1 category, we chose the more intensive of the 2 (eg, transesophageal ECG was categorized as a procedure rather than as an imaging test). We defined a diagnosis as new if the diagnosis was billed on 1 inpatient claim or 2 outpatient claims during the cascade period but not billed in this manner during the 1 year lookback period (eAppendix 3, eTables 3-6 in the Supplement).

We examined the incidence of these cascade events during a 90-day cascade period starting on the day after the AWV test period (ie, 8 days after the AWV). We estimated beneficiary-level spending using allowed charges on Medicare claims for specific cascade event categories (eg, laboratory tests), all cascade-attributable events, and all Medicare expenditures during the 90-day follow-up period (winsorized at the top 0.5%).

#### Patient, Area-Level, and Visit Characteristics

We measured patient and area-level characteristics in the 365-day period preceding the AWV testing period, as well as characteristics associated with the AWVs. We used standard Medicare claims classifications to determine patient characteristics including age, sex, race, disability (as a reason for Medicare enrollment), dual Medicaid enrollment, and Accountable Care Organization participation (using the Medicare Accountable Care Organization Shared Savings Program beneficiary-level file). We used beneficiaries’ residential zip codes to characterize their residential setting (eg, rural vs urban based on Rural-Urban Commuting Area),^19^ US region (based on US Census Bureau^20^), and area-level income (categorized as <200%, 200% to <400%, or ≥400% of the 2014 federal poverty level for a family of 4).^21^ In addition, we used the performing clinician (National Provider Identifier) linked to the AWV to determine clinician specialty (primary care vs other) (eTable 7 in the Supplement). We determined if a given AWV was co-billed as a problem-based visit (using the level 25 billing modifier) because such visits may be more likely to include symptom- or condition-based testing.

### Statistical Analyses

#### Factors Associated With Use of Routine Tests

To determine patient, area-level, and visit characteristics associated with receiving any routine test or 1 of the 3 specific tests, we ran a series of univariate analyses based on age, sex, race, dual Medicaid eligibility, disability, Elixhauser condition count, residential setting, region, area-level income, Accountable Care Organization participation, and specialty of the performing clinician. We then created a multivariate logistic regression model including these patient, area-level, and visit characteristics, as well as hospital referral region random effects, in which the outcome was receipt of any test. Reported *P* values were 2-sided, and statistical significance was set at *P* < .05. We used SAS, version 9.4 (SAS Institute Inc) statistical software for the analyses.

#### Clinician Testing Patterns

Among clinicians who performed at least 10 AWVs in 2014 within our patient cohort, we determined the percentage of their AWVs in which they performed any of the 3 routine tests and each of the 3 tests of interest.

#### Cascade Prevalence

We created unadjusted, beneficiary-level Poisson regression models to estimate cascade event rates and linear regression models to estimate Medicare spending in which the primary predictor was a) receipt of any routine test or b) receipt of a given test.^22^ We then created a series of multivariable regression models to determine event and spending rates, adjusting for the above patient, area-level, and visit characteristics as well as whether or not the visit was co-billed as a problem-based visit (eTable 8 in the Supplement). We included Hospital Referral Region random effects to account for geographic differences in cascade rates. Models for receipt of a given index test additionally controlled for receipt of the other 2 tests within the same testing period. We determined cascade-attributable event rates and spending by subtracting estimates for the comparison group, which represented baseline event rates and spending among Medicare AWV recipients, from estimates for those who received a given test (eAppendix 4 in the Supplement). For estimation of cascade-attributable spending following each index event, we presented these results by event category and excluded hospitalization spending from the main composite cascade-attributable spending outcome given the rarity of these costly events.

#### Sensitivity Analyses

First, recognizing that an index test performed during the cascade period may represent a cascade event, we included ECGs, urinalyses, and thyrotropin tests as cascade events for the respective index tests in a series of sensitivity analyses. Second, other tests performed during AWVs could trigger cascade events that overlap with those examined in this study and could affect our estimates. Therefore, in another sensitivity analysis, we adjusted for patient receipt of 2 other routine tests that may be performed during AWVs: complete blood counts (with and without differential) and metabolic panels (including basic metabolic panel, complete metabolic panel, and liver function tests). These tests were not used as index events in this study because cascade events for these tests were not linkable with sufficient specificity using claims. In addition, while our main analyses were performed on a single cohort of patients, we repeated our main index test and cascade event analyses on 3 separate cohorts, each with prior test- and diagnosis-based exclusions specific to the given index test.

## Results

In our study population of 4 613 032 Medicare beneficiaries, 753 094 beneficiaries received an AWV in 2014, and 75 275 (10.0%) of these AWV recipients met our inclusion criteria (Table 1). These 75 275 AWV recipients had mean (SD) age of 72.6 (6.1) years, and 48 107 (63.9%) were women. Among these healthy asymptomatic beneficiaries, 18.6% (14 017) received at least 1 routine test including an ECG (7.2% [5421]), urinalysis (10.0% [7515]), or thyrotropin (8.7% [6534]). Of all AWV recipients, 12.8% (9631) received 1 of these tests, 4.4% (3319) received 2, and 1.4% (1067) received all 3 tests.

**Table 1.** Patient, Area-Level, and Visit Characteristics Associated With Routine Testing Among Healthy, Asymptomatic Medicare Beneficiaries

Characteristic	No. (%)					Any test, adjusted OR (95%CI)
	ECG (n = 5421)	Urinalysis (n = 7515)	Thyrotropin (n = 6534)	Any test (n = 14 017)	No test (n = 61 258)	
Age, y						
66-74	3892 (71.8)	5483 (73.0)	4735 (72.5)	10 159 (72.5)	42 148 (68.8)	1.69 (1.53-1.86)
75-84	1327 (24.5)	1776 (23.6)	1565 (24.0)	3340 (23.8)	15 449 (25.2)	1.53 (1.38-1.70)
≥85	202 (3.7)	256 (3.4)	234 (3.6)	518 (3.7)	3661 (6.0)	1 [Reference]
Female sex	3403 (62.8)	4690 (62.4)	4468 (68.4)	9027 (64.4)	39 080 (63.8)	1.05 (1.01-1.09)
Race						
White	4918 (90.7)	6836 (91.0)	5969 (91.4)	12 795 (91.3)	56 061 (91.5)	1.32 (1.16-1.49)
Black	139 (2.6)	206 (2.7)	156 (2.4)	347 (2.5)	1525 (2.5)	1 [Reference]
Hispanic	126 (2.3)	154 (2.1)	136 (2.1)	293 (2.1)	1264 (2.1)	1.16 (0.97-1.40)
Other	238 (4.4)	319 (4.2)	273 (4.2)	582 (4.2)	2408 (3.9)	1.30 (1.11-1.51)
Medicaid enrollment	102 (1.9)	129 (1.7)	123 (1.9)	259 (1.9)	1507 (2.5)	0.80 (0.69-0.92)
Disability	167 (3.1)	202 (2.7)	177 (2.7)	413 (3.0)	2275 (3.7)	0.83 (0.74-0.93)
US Census region						
Northeast	2022 (37.3)	1434 (19.1)	1091 (16.7)	3191 (22.8)	12 768 (20.8)	1.28 (1.00-1.62)
Midwest	878 (16.2)	1467 (19.5)	1054 (16.1)	2633 (18.8)	15 610 (25.5)	1 [Reference]
South	1911 (35.3)	3394 (45.2)	3101 (47.5)	5810 (41.5)	18 723 (30.6)	1.61 (1.35-1.92)
West	610 (11.3)	1220 (16.2)	1288 (19.7)	2383 (17.0)	14 157 (23.1)	0.91 (0.73-1.13)
Setting of residence						
Metropolitan	4915 (90.7)	6536 (87.0)	5733 (87.7)	12 187 (86.9)	47 793 (78.0)	1.29 (1.15-1.46)
Micropolitan	302 (5.6)	521 (6.9)	434 (6.6)	1001 (7.1)	7213 (11.8)	0.86 (0.75-0.98)
Suburban	107 (2.0)	254 (3.4)	196 (3.0)	445 (3.2)	3363 (5.5)	0.86 (0.73-1.00)
Rural	97 (1.8)	204 (2.7)	171 (2.6)	384 (2.7)	2889 (4.7)	1 [Reference]
Area-level income, % of the federal poverty level						
0-200	973 (18.0)	1933 (25.7)	1751 (26.8)	3490 (24.9)	18 818 (30.7)	1 [Reference]
201-300	1860 (34.3)	2812 (37.4)	2437 (37.3)	5246 (37.4)	25 067 (40.9)	1.05 (1.00-1.11)
301-400	1457 (26.9)	1730 (23.0)	1462 (22.4)	3277 (23.4)	11 949 (19.5)	1.15 (1.08-1.23)
>400	1131 (20.9)	1040 (13.8)	884 (13.5)	2004 (14.3)	5424 (8.9)	1.26 (1.16-1.37)
Clinician specialty						
Primary care	5077 (93.7)	6854 (91.2)	6138 (93.9)	12 893 (92.0)	53 889 (88.0)	1.59 (1.48-1.70)
Other	344 (6.4)	661 (8.8)	396 (6.1)	1124 (8.0)	7369 (12.0)	1 [Reference]
Accountable Care Organization (patient level)						
Yes	1515 (28.0)	2094 (27.9)	1914 (29.3)	3846 (27.4)	14 939 (24.4)	1.05 (1.00-1.10)
No	3906 (72.1)	5421 (72.1)	4620 (70.7)	10 171 (72.6)	46 319 (75.6)	1 [Reference]
Elixhauser counts, mean (SD)	0.11 (0.35)	0.09 (0.33)	0.09 (0.32)	0.10 (0.34)	0.10 (0.34)	0.99 (0.93-1.04)

Abbreviations: ECG, electrocardiogram; OR, odds ratio.

Patients were more likely to receive any of the 3 routine tests of interest if they were younger (adjusted odds ratio [aOR], 1.69 for ages 66-74 years vs ages ≥85 years [95% CI, 1.53-1.86]), female (aOR, 1.05 [95% CI, 1.01-1.09]), White (aOR, 1.32 compared with Black [95% CI, 1.16-1.49]), lived in urban areas (aOR, 1.29 vs rural [95% CI, 1.15-1.46]), lived in the South (aOR, 1.61 [95% CI, 1.35-1.92]) or Northeast (aOR, 1.28 [95% CI, 1.00-1.62]), or lived in high-income areas (aOR, 1.26 for >400% of the federal poverty level vs <200% of the federal poverty level [95% CI, 1.16-1.37]) (Table 1). Patients were also more likely to receive a low-value test if they saw a primary care physician for their AWV (aOR, 1.59 compared with other specialties [95% CI, 1.48-1.70]). Patients who were dually enrolled in Medicaid (aOR, 0.80 [95% CI, 0.69-0.92]) and with disability (aOR, 0.83 [95% CI, 0.74-0.93]) were less likely to receive any routine test. Most clinicians who ordered these tests did so for less than half of the AWVs that they performed (eFigure 3 in the Supplement).

### Potential Cascade Rates

We found that 23.7% (3322) of the 14 017 beneficiaries who received any routine test had at least 1 potential cascade event, including 15.4% (833) of the 5421 beneficiaries who received ECGs, 13.8% (1039) of the 7515 beneficiaries who received urinalyses, and 6.4% (416) of the 6534 beneficiaries who received thyrotropin tests. Among patients who received any routine test, 13.5% (1890) had a potential cascade laboratory test, 6.6% (927) had a potential cascade imaging test, 3.9% (546) had a potential cascade procedure, 8.6% (1201) had a potential cascade visit, 2.0% (276) had a potential cascade new diagnosis, and 0.3% (37) had a potential cascade hospitalization.

### Cascade Event Rates and Spending

We identified 6.1 (95% CI, 4.8-7.5) cascade-attributable events per 100 beneficiaries in the 90 days following routine ECGs, 5.4 (95% CI, 4.2-6.5) following urinalyses, and −1.0 (95% CI, −1.7 to −0.3) following thyrotropin test (Table 2, Table 3, and Table 4). Excluding hospitalizations, we estimated cascade-attributable spending per beneficiary of $9.62 (95% CI, $6.43-$12.80), $7.46 (95% CI, $5.11-$9.81), and $0.02 (95% CI,$ −1.00 to $1.05), respectively. Receipt of any routine test was associated with cascade-attributable additional spending of $0.64 (95% CI, $0.22-$1.07) for laboratory tests, $1.66 (95% CI, $0.73-$2.59) for imaging tests, $3.38 (95% CI, $1.50-$5.26) for procedures, $2.88 (95% CI, $2.01-$3.75) for visits, and $40.92 (95% CI, % −50.28 to $132.13) for hospitalizations.

**Table 2.** Cascade-Attributable Event Rates and Spending Following Routine Electrocardiogram During an Annual Wellness Visit

Event rate per 100 beneficiaries	Exposure group (n = 5421)	Comparison group (n = 69 854)	Cascade-attributable event rate	Adjusted cascade-attributable event rate (95% CI)^a^
All events^b^	27.4	21.8	5.6	6.12 (4.76-7.49)
Laboratory tests	11.8	11.4	0.4	0.62 (-0.27-1.50)
Imaging	5.0	3.4	1.6	1.58 (1.04-2.12)
Procedures	5.1	3.3	1.8	1.93 (1.42-2.44)
Visits	3.7	2.1	1.6	1.29 (0.86-1.72)
Hospitalizations	<0.20^b^	0.21	NR^b^	NR^b^
New diagnoses	1.8	1.7	0.1	0.47 (0.16-0.78)
Allowable charges per beneficiary, mean (SD), $				
Allowable charges related to cascade events in 90-d period, excluding hospitalizations^c^	23.4	13.2	10.2	9.62 (6.43-12.80)
Total Medicare-allowable charges in 90-d period	3721.9	2722.6	NA	NA

Abbreviations: HRR, hospital referral region; NA, not applicable; NR, not reported.

^a^ This multivariable model includes the following covariates: age, sex, race, Medicaid enrollment, disability, Elixhauser condition count, residential setting, region, area-level income, clinician specialty, Accountable Care Organization status, visit co-billed status, HRR random effects, receipt of urinalysis, and receipt of thyrotropin test.

^b^ Entries with 11 or fewer events are not reported in accordance with the Centers for Medicare and Medicaid Services data use agreement.

^c^ To meet this requirement, we excluded hospitalizations from all event totals in this table.

**Table 3.** Cascade-Attributable Event Rates and Spending Following Routine Urinalysis During an Annual Wellness Visit

Event rate per 100 beneficiaries	Exposure group (n = 7515)	Comparison group (n = 67 760)	Cascade-attributable rate	Adjusted cascade-attributable event rate (95% CI)^a^
All events^b^	29.8	21.2	8.6	5.35 (4.20-6.50)
Laboratory tests	13.4	9.0	4.4	1.73 (1.08-2.39)
Imaging	4.9	3.9	1.0	1.05 (0.54-1.57)
Procedures	2.8	2.0	0.8	0.67 (0.32-1.02)
Visits	7.3	5.1	2.2	1.34 (0.76-1.92)
Hospitalizations	<0.20^b^	0.13	NR^b^	NR^b^
New diagnoses	1.4	1.3	0.2	0.36 (0.07-0.65)
Allowable charges per beneficiary, mean (SD), $				
Allowable charges related to cascade events in 90-d period, excluding hospitalizations^c^	26.8	16.9	9.9	7.46 (5.11-9.81)
Total Medicare-allowable charges in 90-d period	2919.4	2780.7	NA	NA

Abbreviations: ECG, electrocardiogram; HRR, hospital referral region; NA, not applicable; NR, not reported.

^a^ This multivariable model includes the following covariates: age, sex, race, Medicaid enrollment, disability, Elixhauser condition count, residential setting, region, area-level income, clinician specialty, Accountable Care Organization status, visit co-billed status, HRR random effects, receipt of ECG, and receipt of thyrotropin test.

^b^ Entries with 11 or fewer events are not reported in accordance with the Centers for Medicare and Medicaid Services data use agreement.

^c^ To meet this requirement, we excluded hospitalizations from all event totals in this table.

**Table 4.** Cascade-Attributable Event Rates and Spending Following Routine Thyrotropin Test During an Annual Wellness Visit

Event rate per 100 beneficiaries	Exposure group (n = 6534)	Comparison group (n = 68 741)	Cascade-attributable event rate	Adjusted cascade-attributable event rate (95% CI)^a^
All events^b^	9.0	8.8	0.2	−0.99 (−1.72 to −0.26)
Laboratory tests	2.7	2.8	−0.1	−0.43 (−0.74 to −0.12)
Imaging	0.4	0.6	−0.2	−0.21 (−0.39 to −0.04)
Procedures	1.4	1.5	−0.1	−0.26 (−0.54 to 0.01)
Visits	4.1	3.4	0.6	0.21 (−0.29 to 0.70)
Hospitalizations	NR^b^	NR^b^	NR^b^	NR^b^
New diagnoses	0.4	0.4	−0.1	−0.04 (−0.18 to 0.10)
Allowable charges per beneficiary, mean (SD), $				
Allowable charges related to cascade events in 90-d period, excluding hospitalizations^c^	7.6	6.5	1.1	0.02 (−1.00 to 1.05)
Total Medicare-allowable charges in 90-d period	2562.1	2816.7	NA	NA

Abbreviations: ECG, electrocardiogram; HRR, hospital referral region; NA, not applicable; NR, not reported.

^a^ This multivariable model includes the following covariates: age, sex, race, Medicaid enrollment, disability, Elixhauser condition count, residential setting, region, area-level income, clinician specialty, Accountable Care Organization status, visit co-billed status, HRR random effects, receipt of ECG, and receipt of urinalysis.

^b^ Entries with 11 or fewer events are not reported in accordance with the Centers for Medicare and Medicaid Services data use agreement.

^c^ To meet this requirement, we excluded hospitalizations from all event totals in this table.

 Using a rough spending estimate and accounting for the 20% claims sample, the 3 routine tests and cascades associated with ECGs and urinalyses cost $2.1 million in 2014. If we extrapolate costs to the 25 million fee-for-service Medicare beneficiaries eligible for an AWV, assume that 25% of these adults have an AWV in a given year considering increasing AWV adoption rates,^23^ and use estimated testing rates and cascade-attributable costs (excluding hospitalization costs) in this study (with mean charges of $23.11 for ECG, $4.03 for urinalysis, and $22.93 for thyrotropin test), we estimate test and cascade-attributable spending of $34.3 million across these tests, of which $9 million (26%) was associated with the cascades.

In our sensitivity analyses including index tests as cascade events, we estimated fewer cascade events associated with ECGs and more cascade events associated with urinalyses when compared with our main analyses (eTable 9 in the Supplement). In our study cohort, 16.4% (12 306) received a complete blood count test and 20.6% (15 468) received a metabolic panel test. When we additionally adjusted our main results for patient receipt of these tests, our results were largely unchanged (eTable 10 in the Supplement). For our final sensitivity analysis in which we repeated our analyses among test-specific cohorts, we found higher rates of index tests: 10.9% (18 953) of 174 171 AWV recipients received an ECG, 18.2% (84 322) of 463 273 AWV recipients received a urinalysis, and 15.2% (44 472) of 292 773 AWV recipients received a thyrotropin test. In these cohorts, cascade-attributable event rates were directionally consistent though of smaller magnitude (eTable 11 in the Supplement).

## Discussion

In this national Medicare claims analysis, we found that a substantial share of healthy asymptomatic older adults received routine ECGs, urinalyses, or thyrotropin tests during their AWVs and that these low-value ECGs and urinalyses were associated with cascade events.

We found that 19% of beneficiaries received routine testing during their AWV, a visit for which Medicare requires a series of evidence-based preventive and routine care for aging adults^12^ but does not cover routine testing. What’s more, the visit itself is widely advertised as free to patients, and our findings substantiate anecdotal evidence of “surprise bills” for these tests and their cascades (even with partial coverage for tests and cascades through Medicare and especially for those without supplemental coverage).^24^ These testing rates were somewhat lower than the 25% of self-reported annual physical examinations with screening urinalysis and the 11% with screening ECG estimated in earlier studies using US national survey data and studies from Ontario, Canada, which may reflect the unique function of AWVs or our exclusion of patients with indications appropriate for testing.^9,10,13,25^

Among AWV recipients, routine tests were more commonly performed for younger Medicare beneficiaries, perhaps reflecting the perception that individuals with greater life expectancy may have more use for a screening test. They were also more common among urban-dwelling^14^ adults and those in high-income areas,^26^ similar to other studies on low-value care. White beneficiaries were also more likely to receive a routine test (previous research has found a mixed relationship between race and receipt of low-value services that varies by the type of service^27,28^).

When estimating rates of cascades, we found that ECGs and urinalyses were associated with cascades. Our ECG results are consistent with prior estimates of cascades following routine ECGs performed among Medicare beneficiaries for preoperative evaluation before cataract surgery^14^ and corroborate findings that ECGs during wellness visits in Canada were associated with greater risk of subsequent events.^13,29^ Our findings of cascades following urinalyses align with prior work showing that these tests often show ambiguous or false-positive results.^30^ While routine thyrotropin tests were common, only 6% of patients who received them had any potential cascade event, and they had no additional cascade-attributable events relative to the comparison group in the subsequent 90 days.

These cascades carried modest additional costs per person that nonetheless may quickly accrue across Medicare beneficiaries and represent meaningful out-of-pocket payments for patients. While our analysis focuses on cascade costs observable in Parts A and B Medicare claims data, we note that the cascades we describe may carry substantial direct and indirect costs for patients and clinicians as well.^6,15,16,31,32,33^ These costs include treatment burden (eg, time and travel costs) and psychological and physical harms for patients as well as frustration and anxiety for clinicians.^15,32^

This national study of Medicare beneficiaries is, to our knowledge, the first to examine routine testing and cascades associated with AWVs and to estimate cascades associated with urinalysis and thyrotropin tests while developing and assessing a comprehensive, literature-based inventory of potential cascade events.

### Limitations

We acknowledge limitations similar to other claims-based studies of low-value care. For instance, we did not have clinical details, such as test results and physical examination findings, to confirm intentions behind billed services. However, we identified index tests as low value through rigorous selection of a patient cohort that had no prior or visit-associated condition or symptom nor a testing history that would suggest a nonroutine indication for these tests.^34^ We note that we cannot definitively link index events to cascade events and that unmeasured confounders may contribute to our findings. For instance, despite careful cohort selection and controlling for key confounders, including patient sociodemographic characteristics, comorbidities, and the AWV co-occurring with a problem-based visit, those with suspected but undocumented conditions may have been more likely both to receive a routine test and to experience a potential cascade. Conversely, some patients may receive their routine testing during other visits, which would bias our results to the null, although we address this point by excluding patients who had an index test in the past 6 months. In addition, we note that, while some cascades captured in this study may have contributed to improved health, there is clear evidence codified in society recommendations that, on average, these routine tests offer little benefit and the potential for harms.^1,2,3,4,5,6,7,8^

## Conclusions

In this national Medicare claims-based study, a substantial minority of healthy asymptomatic older adults received a routine ECG, urinalysis, or thyrotropin test during their AWVs. The ECGs and urinalyses were associated with care cascades including laboratory tests, imaging tests, and procedures. Across index tests, cascade laboratory tests and specialist visits were the most common cascade events, while cascade-attributable costs were highest for procedures and specialist visits. Taken together with prior work, our results suggest that even low-value services with small upfront costs may generate cascades that may present both financial and nonfinancial burdens to patients, clinicians, health systems, and payers.^15^

This study may be used in several ways. First, payers and health systems might apply the detailed cascade event definitions we present here to measure the use of these low-value tests and their cascades in their own patient populations. Second, these estimates of routine testing and cascades provide key information for the Centers for Medicare and Medicaid Services on the unintended consequences associated with AWVs that can inform future policies on how these visits are paid for and explained to beneficiaries.^23^ Third, the estimates may be used by health systems and payers to design interventions to reduce low-value testing and its consequences.^35^ For example, cascade rates for ECGs and urinalyses can be incorporated into point-of-care tools to guide clinicians on test ordering^36^ and to quantify the consequences of these tests in conversations with patients.^15,34,35^ It will then be important to test potential interventions to reduce both low-value services and the cascades that follow.

## Notes

Supplement. **eAppendix 1.** Relevant Choosing Wisely Recommendations **eFigure 1.** Study Timeline **eFigure 2.** Study Cohort Flow Diagram **eTable 1.** Excluding Diagnoses **eAppendix 2.** Index Event Definition **eTable 2.** Index Test Codes **eAppendix 3.** Cascade Event Definition **eTable 3.** Cascade Laboratory Tests, Imaging Tests, and Procedures **eTable 4.** Cascade Visits **eTable 5.** Cascade Hospitalizations **eTable 6.** Cascade New Diagnoses **eTable 7.** Primary Care Physician Specialty Codes **eAppendix 4.** Cascade-Attributable Event Analysis **eTable 8.** Prevalence of Routine Tests Among Annual Wellness Visits, by Problem Based Visit Co-Billed Status **eFigure 3.** Physician Test Ordering Patterns **eTable 9.** Cascade-Attributable Event Rates and Spending Following Routine Testing During Annual Wellness Visits, Sensitivity Analysis in Which Index Tests Included as Cascade Events **eTable 10.** Cascade-Attributable Event Rates and Spending Following Routine Testing During Annual Wellness Visits, Sensitivity Analysis Adjusted for Receipt of Other Routine Tests **eTable 11.** Index Test and Cascade-Attributable Event Rates Following Routine Testing During Annual Wellness Visits, Sensitivity Analysis Using Test-Specific Cohorts **eReferences**
