# Supplementary material for: Assessment of Prevalence and Cost of Care Cascades After Routine Testing During the Medicare Annual Wellness Visit
Source: JAMA Netw Open. 2020 Dec 11;3(12):e2029891. doi: 10.1001/jamanetworkopen.2020.29891 (PMC7733154; doi:10.1001/jamanetworkopen.2020.29891)
Supplement: Supplement. — eAppendix 1. Relevant Choosing Wisely Recommendations eFigure 1. Study Timeline eFigure 2. Study Cohort Flow Diagram eTable 1. Excluding Diagnoses eAppendix 2. Index Event Definition eTable 2. Index Test Codes eAppendix 3. Cascade Event Definition eTable 3. Cascade Laboratory Tests, Imaging Tests, and Procedures eTable 4. Cascade Visits eTable 5. Cascade Hospitalizations eTable 6. Cascade New Diagnoses eTable 7. Primary Care Physician Specialty Codes eAppendix 4. Cascade-Attributable Event Analysis eTable 8. Prevalence of Routine Tests Among Annual Wellness Visits, by Problem Based Visit Co-Billed Status eFigure 3. Physician Test Ordering Patterns eTable 9. Cascade-Attributable Event Rates and Spending Following Routine Testing During Annual Wellness Visits, Sensitivity Analysis in Which Index Tests Included as Cascade Events eTable 10. Cascade-Attributable Event Rates and Spending Following Routine Testing During Annual Wellness Visits, Sensitivity Analysis Adjusted for Receipt of Other Routine Tests eTable 11. Index Test and Cascade-Attributable Event Rates Following Routine Testing During Annual Wellness Visits, Sensitivity Analysis Using Test-Specific Cohorts eReferences [file jamanetwopen-e2029891-s001.pdf]

Supplementary Online Content

Ganguli I, Lupo C, Mainor AJ, Wang Q, Orav EJ, Rosenthal MB, Sequist TD, Colla CH. Assessment of prevalence and cost of care cascades after routine testing during the Medicare annual wellness visit. *JAMA Netw Open.* 2020;3(12):e2029891. doi:10.1001/jamanetworkopen.2020.29891

eAppendix 1. Relevant Choosing Wisely Recommendations

eFigure 1. Study Timeline

eFigure 2. Study Cohort Flow Diagram

eTable 1. Excluding Diagnoses

eAppendix 2. Index Event Definition

eTable 2. Index Test Codes

eAppendix 3. Cascade Event Definition

eTable 3. Cascade Laboratory Tests, Imaging Tests, and Procedures

eTable 4. Cascade Visits

eTable 5. Cascade Hospitalizations

eTable 6. Cascade New Diagnoses

eTable 7. Primary Care Physician Specialty Codes

eAppendix 4. Cascade-Attributable Event Analysis

eTable 8. Prevalence of Routine Tests Among Annual Wellness Visits, by Problem Based Visit Co-Billed Status

eFigure 3. Physician Test Ordering Patterns

eTable 9. Cascade-Attributable Event Rates and Spending Following Routine Testing During Annual Wellness Visits, Sensitivity Analysis in Which Index Tests Included as Cascade Events

eTable 10. Cascade-Attributable Event Rates and Spending Following Routine Testing During Annual Wellness Visits, Sensitivity Analysis Adjusted for Receipt of Other Routine Tests

eTable 11. Index Test and Cascade-Attributable Event Rates Following Routine Testing During Annual Wellness Visits, Sensitivity Analysis Using Test-Specific Cohorts

eReferences

© 2020 Ganguli I et al. *JAMA Network Open*.

eAppendix 1. Relevant Choosing Wisely recommendations

Society of General Internal Medicine: **For asymptomatic adults without a chronic medical condition, mental health problem, or other health concern, don't routinely perform annual general health checks that include a comprehensive physical examination and lab testing. Adults should talk with a trusted doctor about how often they should be seen to maintain an effective doctor-patient relationship, attend to preventive care, and facilitate timely recognition of new problems.**
Visit intervals should be based on specific concerns, chronic conditions, or prevention strategies based on the best available evidence, tailored to age and risk. A general health check may help to foster a trusting relationship between a doctor and patient. It may also provide an opportunity for preventive counseling and screening. However, it is not always necessary to have a general health check every year. In contrast to office visits for acute illness, specific evidence-based preventive strategies, or chronic care management such as treatment of high blood pressure, annually scheduled general health checks, including the "health maintenance" visit, have not been shown to reduce morbidity, hospitalizations, or mortality, and may increase the frequency of non-evidence based testing.[1]

Health Checkups: Tests and screenings can cause problems.
Most people should only have a test or screening if they have symptoms or risks factors. One problem is getting a false-positive result. These false alarms can cause anxiety, and unnecessary follow-up tests and treatments. For example, a false-positive blood test can result in a biopsy. An ECG that is not interpreted correctly may lead to another test that exposes you to radiation. Or you might get a procedure that has a risk of heart attack or death in two patients for every 100 who get the test.[2]

© 2020 Ganguli I et al. *JAMA Network Open*.

eMethods

eFigure 1. Study timeline

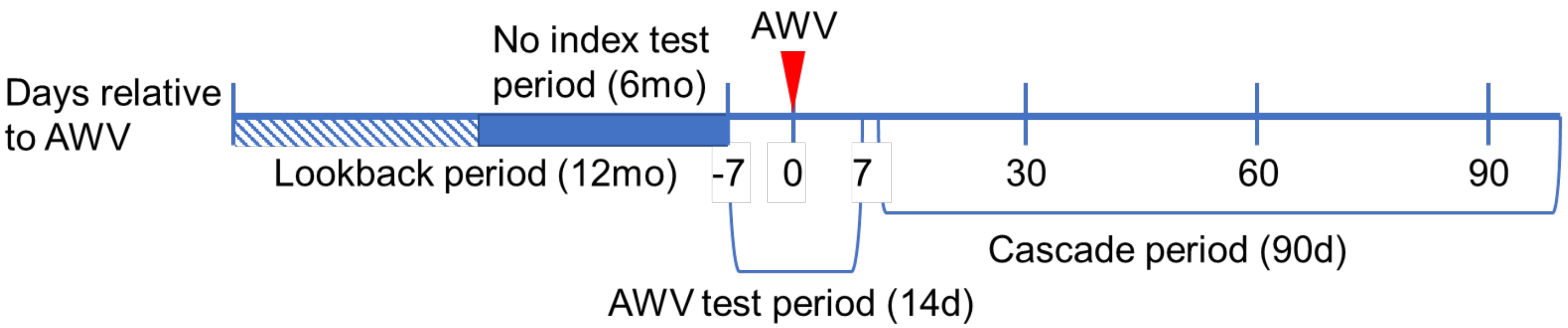

Abbreviation: AWV, Annual Wellness Visit

© 2020 Ganguli I et al. *JAMA Network Open*.

eFigure 2. Study cohort flow diagram

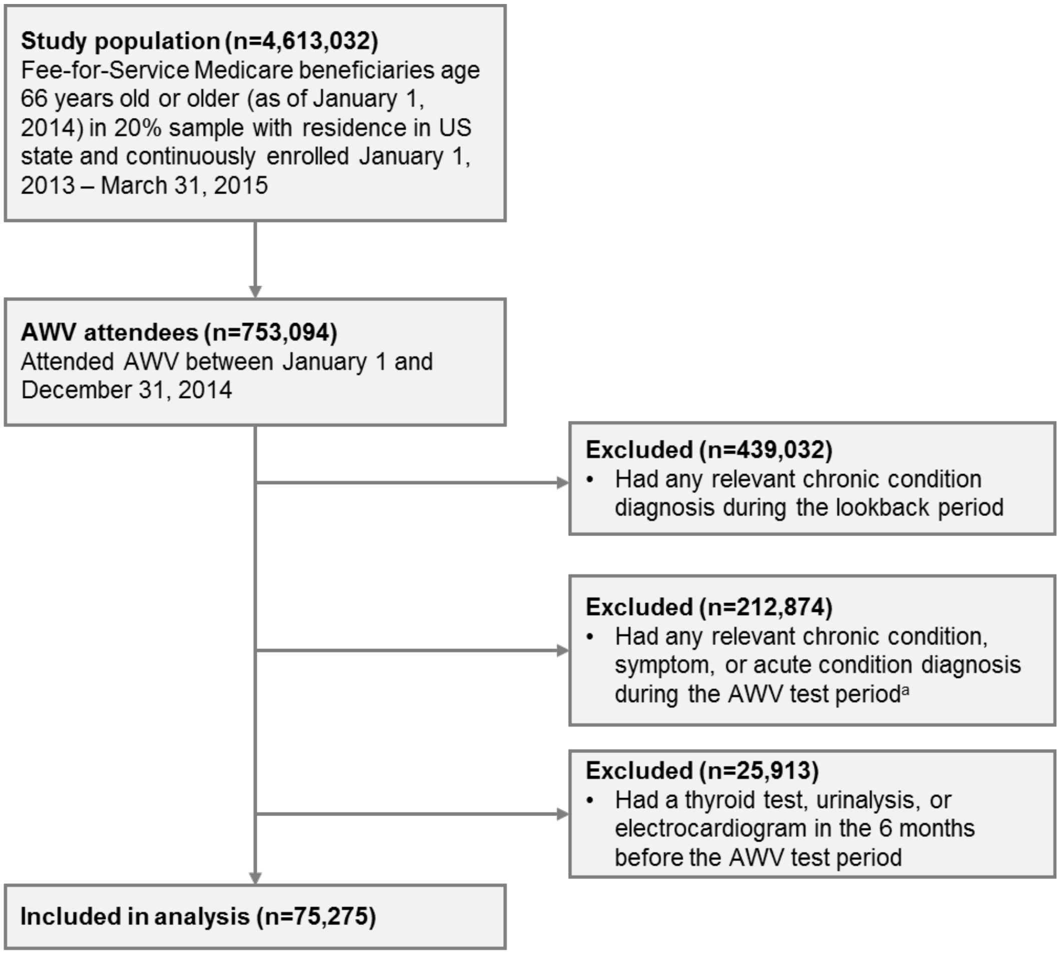

Abbreviation: AWV, Annual Wellness Visit
[a] AWV test period = 7 days before or after AWV

Definition of Annual Wellness Visit (AWV) – Yearly appointment with primary care provider to create or update personalized prevention plan and perform a health risk assessment. One AWV is covered by Medicare every 12 months for beneficiaries who are no longer within 12 months after the beneficiary's Medicare Part B benefits eligibility date.[2]

Diagnosis-based exclusions - We excluded beneficiaries with a relevant chronic condition if the diagnosis code was present in one inpatient or two outpatient claims in the prior year OR in any claim during the AWV test period. We excluded beneficiaries with a relevant symptom or acute condition if the diagnosis code was present in any claim during the AWV test period. See eTable 1 for codes.

eTable 1. Excluding diagnosis codes

Test	Diagnosis type	Diagnoses	ICD-9 codes
**ECG**	Chronic conditions	Hypertension (CCW)	362.11, 401.0-1, 401.9, 402.00-01, 402.10-11, 402.90-91, 403.00-01, 403.10-11, 403.90-91, 404.00-03, 404.10-13-93, 405.01, 405.09, 405.11, 405.19, 405.91, 405.99, 437.2
		Ischemic heart disease (CCW)	410, 410.0-01, 410.00-02, 410.10-2, 410.20-22, 410.30-32, 410.40-42, 410.50-52, 410.60-62, 410.70-72, 410.80-82, 410.90-92, 411.0-1, 411.81, 411.89, 412, 413.0-1, 413.9, 414.00-07, 414.10-12,414.19, 414.2-4, 414.8-9
		Heart failure (CCW)	398.91, 402.01, 402.11, 402.91, 404.01, 404.03, 404.11, 404.13, 404.91, 404.93, 428.0-1, 428.9, 428.20-23, 428.30-33, 428.40-43
		Valvular disease (CCW)	394.0-2, 394.9, 395.0-2, 395.9, 396.0-3, 396.8-9, 397.1, 397.9, 421.2, 424.0-3
		Arrythymia (CCW)	426.0, 426.2-7, 426.10-13, 426.50-4, 426.81-2, 426.89, 426.9, 427.0-2, 427.9, 427.31-2, 427.41-2, 427.60-1, 427.69, 427.81, 427.89, V45.01
	Symptoms and acute conditions	Chest pain	786.50-2, 786.59
		Dyspnea	786.00-7, 786.09
		Hypotension	458.9
		Bradycardia	427.89
		Nausea	787.01-03
		Unconsciousness, syncope	780.09, 780.2
		Dizziness	780.4
		Tachycardia, palpitations	785.0-1
		Lower extremity edema	782.3
		Nonspecific abnormal ECG	794.31
**UA**	Chronic conditions	Acute/chronic kidney disease (CCW)	016.00-06, 095.4, 189.0, 189.9, 223.0, 236.91, 249.40-41, 250.40-43, 271.4, 274.10, 283.11, 403.01, 403.11, 403.91, 404.02-03, 404.12-13, 404.92-93, 440.1, 442.1, 572.4, 580.0, 580.4, 580.81, 580.89, 580.9, 581.0-3, 581.81, 581.89, 581.9, 582.0-2, 582.4, 582.81, 582.89, 582.9, 583.0-2, 583.4, 583.6-7, 583.81, 583.89, 583.9, 584.5-9, 585.1-6, 585.9, 586, 587, 588.0-1, 588.81, 588.89, 588.9, 591, 753.12-17, 753.19-23, 753.29, 794.4
		Hypertension	As above
		Nephrolithiasis, ureteral calculus	592.0-1
		Benign prostatic hyperplasia	600.00-1, 600.10-1, 600.20-1, 600.3, 600.90-1
		Glomerulonephrosis, glomerulonephritis, neurogenic bladder, indwelling urinary catheter, spinal cord injury, prior renal transplant	581.1, 582.1, 596.54, 57.94, 952.0-9, V42.0

	Symptoms and acute conditions	Flank pain/renal colic	788.0, 789.0
		Urinary tract infection (including pyelonephritis, cystitis)	590.80, 595.0, 599.0, 112.2
		Hematuria, hematospermia, dysuria, urinary frequency, excessive urination, nocturia, urinary urgency, hesitancy, pus in urine, proteinuria	599.70, 608.82, 788.1, 788.41, 788.43, 788.63-4, 791.9, 791.0
		Urethritis, prostatitis, epididymitis, pelvic infection	597.80, 601.9, 604.90, 614.9
**TSH**	Chronic conditions	Goiter, thyrotoxicosis, hyperthyroidism	240.0, 240.9, 241.0-1, 241.9, 242.00-01, 242.10-11, 242.20-21, 242.30-31, 242.40-41, 242.80-81, 242.90-91
		Hypothyroidism	243, 244.0-3, 244.8-9
		Thyroiditis	245.0-4, 245.8-9
		Unspecified disorder of thyroid	246.0-3, 246.8-9
		Thyroid neoplasm	193, 198.89, 226, 237.4,
		Personal history medullary thyroid cancer, FHx other malignancy; personal, family history multiple endocrine neoplasia syndrome	V10.87, V16.8, V18.11, 258.02-03
		Diabetes (CCW)	249.00-01, 249.10-11, 249.20-21, 249.30-31, 249.40-41, 249.50-51, 249.60-61, 249.70-71, 249.80-81, 249.90-91, 250.00-03, 250.10-13, 250.20-23, 250.30-33, 250.40-43, 250.50-53, 250.60-63, 250.70-73, 250.80-83, 250.90-93, 357.2, 362.01-06, 366.41
		Anemia (CCW)	280.0-1, 280.8-9, 281.0-4, 281.8-9, 282.0-3, 282.40-47, 282.49, 282.5, 282.60-64, 282.68-69, 282.7-9, 283.0, 283.10-11, 283.19, 283.2, 283.9, 284.01, 284.09, 284.1-12, 284.19, 284.2, 284.81, 284.89, 284.9, 285.0-1, 285.21-22, 285.29, 285.3, 285.8, 285.9
		History of irradiation	V15.3, E962.3
		Down's syndrome	758.0
	Symptoms and acute conditions	Muscle weakness, myalgia, chronic fatigue, other malaise and fatigue	728.87, 729.1, 780.71, 780.79
		Exophthalmos, opththalmoplegia	376.22
		Abnormal weight gain/loss	783.1-2
		Excessive sweating	780.8
		Constipation	564.00-09
		Neck lump, dysphonia, dysphagia	784.2, 784.42, 787.20
		Tachycardia, palpitations	427.31, 785.0-1
		Abnormal results of thyroid function studies	794.5
		Heat or cold intolerance	780.99

		Hypertension (CCW)	As above
		Depression (CCW)	296.20, 296.21, 296.22, 296.23, 296.24, 296.25, 296.26, 296.30, 296.31, 296.32, 296.33, 296.34, 296.35, 296.36, 296.51, 296.52, 296.53, 296.54, 296.55, 296.56, 296.60, 296.61, 296.62, 296.63, 296.64, 296.65, 296.66, 296.89, 298.0, 300.4, 309.1, 311
		Alzheimer's and related disorders or senile dementia (CCW)	331.0, 331.11, 331.19, 331.2, 331.7, 290.0, 290.10, 290.11, 290.12, 290.13, 290.20, 290.21, 290.3, 290.40, 290.41, 290.42, 290.43, 294.0, 294.10, 294.11, 294.20, 294.21, 294.8, 797

Abbreviations: ECG, electrocardiogram; UA, urinalysis; TSH, thyrotropin test (also known as thyroid stimulating hormone test). CCW, Medicare Chronic Conditions Warehouse. CCW indicates these diagnosis codes were based on CCW condition categories (https://www2.ccwdata.org/web/guest/condition-categories).

eAppendix 2. Index event definition

Definition of index event - Routine lab tests in conjunction with a patient's annual wellness visit (AWV). Defined as routine tests performed within 7 days before or after the AWV.

eTable 2. Index test codes

Test Type	CPT Code
ECG	93000, 93005, 93010, G0403-5
UA	81000-3
TSH	84443

eAppendix 3. Cascade event definitions

Definition of cascade events - Follow-up testing, procedures, visits, hospitalizations, and new diagnoses which plausibly follow from the index event that were measured during the cascade period.

eTable 3. Cascade laboratory tests, imaging tests, and procedures

A. Electrocardiogram

Cascade category	Event	Codes
**Laboratory tests**	Electrolytes	80048, 80051, 80053
	Brain natriuretic peptide	83880
	Troponin	84484
**Imaging**	Rhythm tracing / monitoring	93040-2, 93224-9, 93268, 93270-2, 93278
	Echocardiogram	93303-4, 93306-8, 93313, 93320-1, 93325
	Cardiac MRI, CT	75557, 75561, 75565, 75571-4
	Nuclear medicine imaging	78428, 78499, 78472, 78454, 78459, 78466, 78468-9, 78481, 78494, 78496

Cascade category	Event	Codes
**Procedures**	Electrophysiology procedure	33202-3, 33206-8, 33210-8, 33220-31, 33233-8, 33240-1, 33243-4, 33249, 33250-1, 33254-9, 33261-6, 33270-3, 33282, 33284, 92953, 92960-1, 92970-1, 93260-1, 93279-99, 93600, 93602-3, 93609-10, 93612-3, 93615-6, 93618-24, 93640-2, 93644, 93650-4, 93655-7, 93660, 93662, E0616
	Valve repair/replacement	33361-9, 33400-1, 33403, 33405-6, 33410-8, 33420, 33422, 33425-7, 33430, 33460, 33463-5, 33474-5, 33478, 92986-7, 92990, 92992, 0343T
	Percutaneous coronary angioplasty/cardiac catheterization	92920-1, 92924-5, 92928-9, 92933-4, 92937-8, 92941, 92943-4, 92973, 92975, 92977, 92980-2, 92984, 92995-6
	Coronary artery repair and bypass graft surgery	33500-5, 33507, 33510-4, 33516-9, 33521-3, 33530, 33533-6, 33542, 33545, 33548, 33572, 92978-9
	Stress test	75559, 75563, 78414, 7845-3, 78473, 78483, 78491-2, 93015-8, 93024
	Transesophageal echocardiography	93312, 93314-8, 93350-2, 93355

B. Urinalysis

Cascade category	Event	Codes
**Laboratory tests**	Electrolytes and kidney function tests	80051, 80069, 82040, 84300, 84132-3, 84295, 84520
	Urine culture	87077, 87081, 87086, 87088, 87184-6
	Creatine kinase	82550
	Metanephrines	83835
	Protein electrophoresis	82784, 84155-6, 84165-6, 86334, 83883
	Antibody tests (ANCA, ANA, dsDNA, ASM)	86021, 86038-9, 86225, 86235, 86255-6
	Tests for infection (including tuberculosis, chlamydia, gonorrhea)	86481, 86580, 87206, 87491, 87591, 88312-3
	Urine cytology	88104, 88108, 88112
**Imaging**	Renal angiography	36251-4
	Pelvic CT, MRI	72192-7
	Abdomen and Abdomen/pelvic CT, MRI	74150, 74160, 74170, 74176-8, 74181-3
	Urinary tract X-ray	74420
	Retroperitoneal and pelvic ultrasound	76770, 76775, 76856-7
**Procedures**	Liver biopsy	47000-1, 47100, 47379, 47700
	Cholecystectomy	47562-3
	Retroperitoneal, renal biopsy	49180, 50200
	Nephrectomy	50543, 50545, 50548
	Renal laparoscopy	50549
	Lithotripsy	50590
	Litholapaxy	52317-8
	Renal ablation	50592
	Cystourethroscopy	52000, 52005, 52204, 52224, 52351-3
	Imaging-guided procedure	76942, 77012, 77021

C. Thyrotropin test

Cascade category	Event	Codes
**Laboratory tests**	Thyroid function test	84436, 84439, 84479
	Parathyroid hormone	83970

	Triiodothyronine T3	84480-1
	Microsomal antibody	86376
	Thyroglobulin antibody	86800
**Imaging**	CT soft tissue neck	70490-2
	Thyroid ultrasound	76536
	Thyroid nuclear medicine	78012-14
	Parathyroid planar imaging	78070-2
**Procedures**	Thyroid biopsy with imaging	10022, 60100, 76940, 76942, 77012, 77021
	Thyroidectomy	60210, 60212, 60220, 60225, 60240, 60252, 60254, 60260, 60270-1
	Aspiration of thyroid cyst	60300
	Other procedure of the endocrine system	60699
	Clinical brachytherapy radiation treatment	77750
	Radiopharmaceutical therapy oral administration	79005

eTable 4. Cascade visits

Index Test	Specialties	Codes
**ECG**	Cardiology, Cardiac Electrophysiology, Interventional Cardiology, Pulmonology, Cardiac Surgery, Thoracic Surgery	6, 21, 29, 33, 78, C3
**UA**	Hematology, Hematology/Oncology, Medical Oncology, Surgical Oncology, Radiational Oncology, Gynecologic Oncology, Pulmonology, Gastroenterology, Urology, Nephrology, Infectious Disease, Rheumatology, Thoracic Surgery, General/Colorectal Surgery	2, 10, 28, 29, 33, 34, 39, 44, 66, 82, 83, 90-92, 98
**TSH**	Hematology/Oncology, Medical Oncology, Surgical Oncology, Radiational Oncology, Endocrinology, General Surgery, Otolaryngology	2, 4, 46, 83, 90-92

eTable 5. Cascade hospitalizations

A. Electrocardiogram

Hospitalization diagnosis	Primary diagnosis codes
Structural heart disease	395.2, 396.3, 423.9, 424.0-1, 428.0, 428.21, 428.23, 428.31, 428.33, 428.43, 429.83
Ischemic heart disease	410.11, 410.21, 410.41, 410.71-2, 410.81, 410.91, 411.1, 414.00-1
Arrhythmia	426.0, 426.4, 426.6, 427.1, 426.12-3, 427.31-2, 427.81, 427.89
Abnormal cardiac study	746.89, 794.39

B. Urinalysis

Hospitalization diagnosis	Primary diagnosis codes
Benign mass	220, 211.3, 235.2, 239.5, 620.2, 621.4, 751.0
Malignancy	152.0, 153.3-4, 153.9-4.1, 156.0, 157.0-2, 157.9, 180.9, 182.0, 183.0, 184.4, 188.9-9.0, 197.6, 198.7, 198.82, 209.03
Genitourinary (GU) infection/inflammation	446.4, 599.0, 614.3, 590.80
Calculus/obstruction	592.0-1, 593.4, 593.9, 574.10, 574.50
Abnormal GU study	620.9, 596.89

C. Thyrotropin test

Hospitalization diagnosis	Primary diagnosis codes
Panhypopituitarism	253.2
Abnormal thyroid study	253.6

eTable 6. Cascade new diagnoses (defined as a diagnosis that appears in one inpatient or two outpatient claims during the cascade period but does NOT appear in in one inpatient or two outpatient claims during the year prior to the index event)

A. Electrocardiogram

Cascade new diagnosis	ICD-9 Codes
Ischemic heart disease	410, 410.0-1, 410.00-2, 410.10-2, 410.20-2, 410.30-2, 410.40-2, 410.50-2, 410.60-2, 410.70-2, 410.80-2, 410.90-2, 411.0-1, 411.81, 411.89, 412, 413.0-1, 413.9, 414.00-7, 414.10-2,414.19, 414.2-4, 414.8-9
Heart failure	398.91, 402.01, 402.11, 402.91, 404.01, 404.03, 404.11, 404.13, 404.91, 404.93, 428.0-1, 428.9, 428.20-3, 428.30-3, 428.40-3
Valvular/structural disease	394.0-2, 394.9, 395.0-2, 395.9, 396.0-3, 396.8-9, 397.0-1, 397.9, 403.01, 403.91, 421.2, 424.0-3
Arrhythmia	426.0, 426.10-13, 426.2-7, 426.50-4, 426.81-2, 426.89, 426.9, 427.0-2, 427.9, 427.31-2, 427.41-2, 427.60-1, 427.69, 427.81, 427.89

B. Urinalysis

Cascade new diagnosis	ICD-9 codes
Tuberculosis, Chlamydia, Gonorrhea	016.00-06, 799.8, 980
Gastrointestinal malignancy (colon, liver, pancreatic)	153.0-4.1, 155.0, 155.1, 157.9, 230.3-4
Genitourinary (GU) tract malignancy	188.9, 189.0, 189.9, 194.0, 236.91, 237.2
Benign gastrointestinal mass	211.1, 211.3, 211.5, 211.6, 577.2
Benign genitourinary tract mass	223.0, 220, 227.0
Hemangioma	228.00, 228.02, 228.04, 228.09
Splenic cyst	289.59
Adrenal disorder	255.8
Renal glycosuria	271.4
Monoclonal paraproteinemia	273.1
Amyloidosis	277.30, 277.39
Renal disease	403.01, 403.11, 403.91, 404.01-3, 404.11-3, 404.91-3, 446.21, 580.0, 580.4, 580.81, 580.89, 580.9-13, 581.81, 581.89, 581.9-2.2, 582.4, 582.81, 582.89, 582.9-3.2, 583.4, 583.6-7, 583.81, 583.89, 583.9, 585.9, 587, 588.89, 588.9, 591
Diverticulosis	562.10
Liver disease	571.0, 571.2, 571.5, 571.8
Hepatitis	573.3
Gallbladder disease	574.1-2, 574.7, 574.9
Nephrolithiasis, ureteral calculus	592.0-1
Renal cyst	593.2, 753.10, 753.12-14, 753.16-7, 753.19
GU inflammation / infection (including cystitis, pyelonephritis, urethritis, prostatitis, orchitis/epididymitis, pelvic inflammatory disease)	590.8, 595.0, 597.80, 599.0, 601.9, 604.90, 614.90
Hematuria	599.70
Proteinuria	791.0
Prostate cyst	600.3
Gastrointestinal vessel anomaly	747.61
Meckel's diverticulum	751.0
Liver cyst	751.60-2, 573.8
Renal dysplasia	753.15, 753.20-2, 753.29
Ureterocele	753.23
Lymph node enlargement	785.6
Pulmonary nodule	793.11, 793.19
Abnormal kidney function study	794.4

C. Thyrotropin test

Cascade new diagnosis	ICD-9 codes
Thyroid cancer	193
Thyroid nodules, goiter	226, 240.0, 241.0, 241.1, 241.9, 242.20-1, 242.30-1, 242.40-1, 246.1
Pituitary adenoma	227.3
Endocrine tumor	237.4
Hypothyroidism	243, 244.0, 244.8-9
Hyperthyroidism	242.00, 242.01, 242.10, 242.80-81, 242.90-91
Thyroiditis	245.0-4, 245.8-9
Other thyroid disorder	246.0, 246.8-9
Euthyroid sick syndrome	790.94
Thyroid cyst	246.2
Thryoid hemorrhage/infarction	246.3
Panhypopituitarism	253.2
Hyperparathyroidism	252.0
Hypoparathyroidism	252.1
Multiple endocrine neoplasia	258.01-3
Abnormal thyroid function study	794.5

Covariates

eTable 7: Primary care physician specialty codes

Specialty	Specialty code
General practice	01
Family practice	08
Internal medicine	11
Pediatric medicine	37
Geriatric medicine	38
Preventive medicine	84

Specialty codes used to identify specialty of physician providing Annual Wellness Visit. All other specialties as well as physician assistants and nurse practitioners (for whom specialties are not listed in Medicare data) were included as "other."

eAppendix 4. Cascade-attributable event analysis

Estimation of cascade-attributable event rates - We estimated event rates for the exposure and comparison groups using the parameters of the Poisson model to calculate the predicted mean rate for an average patient on the log-scale, and then back-transformed to get adjusted event rates for each group. These two rates were then subtracted to get the rate difference, or adjusted cascade-attributable event rate, for which confidence intervals were calculated based on the p-value of the Intervention effect in the Poisson model (using a standard error consistent with the original p-value and the estimated rate difference).

eResults

eTable 8. Prevalence of routine tests among Annual Wellness Visits, by problem based visit co-billed status

Routine test	AWV co-billed with problem based visit	
	Yes, %<br>N=17,270	No, %<br>N=58,005
Any test	27.7	15.9
ECG	11.3	6.0
UA	15.9	8.2
TSH	13.9	7.1

eFigure 3. Physician test ordering patterns

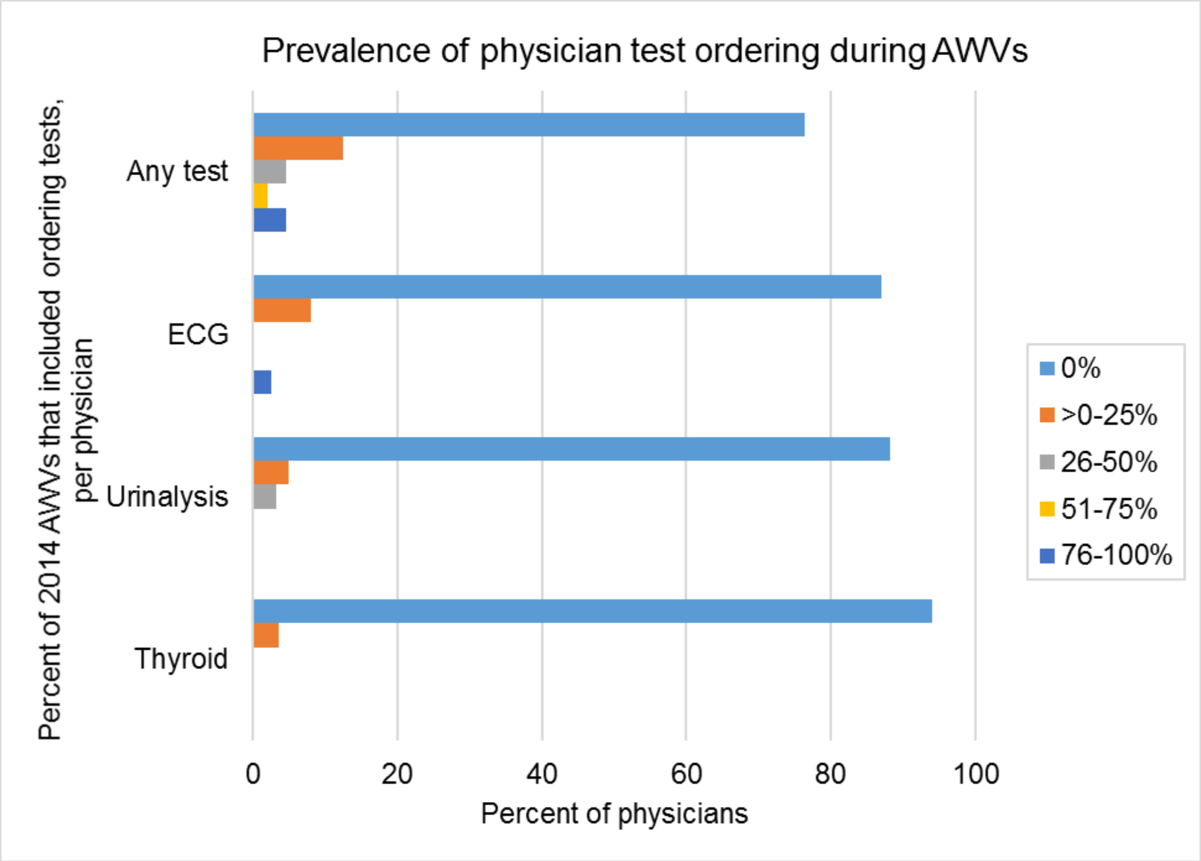

Test ordering patterns among the 599 clinicians (defined by National Provider Identifier) who performed at least 10 Annual Wellness Visits in 2014.

eTable 9. Cascade-Attributable Event Rates and Spending Following Routine Testing During Annual Wellness Visits, Sensitivity Analysis in Which Index Tests Included as Cascade Events

Event rate per 100 beneficiaries	Adjusted cascade-attributable event rate (95%CI)		
	**ECG**	**UA**	**TSH**
All events	4.92 (3.34, 6.49)	6.41 (5.07, 7.75)	-3.72 (-4.59, -2.85)
Laboratory tests	0.62 (-0.27, 1.50)	2.86 (1.90, 3.81)	-2.72 (-3.27, -2.17)
Imaging	0.50 (-0.45, 1.44)	1.05 (0.54, 1.57)	-0.21 (-0.39, -0.04)
Procedures	1.93 (1.42, 2.44)	0.67 (0.32, 1.02)	-0.26 (-0.54, 0.01)
Visits	1.29 (0.86, 1.72)	1.34 (0.76, 1.92)	0.21 (-0.29, 0.70)
New diagnoses	0.47 (0.16, 0.78)	0.36 (0.07, 0.65)	-0.04 (-0.18, 0.10)
Allowable charges related to cascade events in 90-day period excluding hospitalizations, $ mean (SD)[c]	9.48 (6.23, 12.72)	7.51 (5.15, 9.87)	-0.73 (-1.78, 0.32)

Results adjusted for patient age, sex, race, Medicaid, disability, Elixhauser condition count, residential setting, region, area-level income, ACO participation, specialty of clinician performing visit, visit co-billed status, and HRR random effects (as in main analyses).

eTable 10. Cascade-Attributable Event Rates and Spending Following Routine Testing During Annual Wellness Visits, Sensitivity Analysis Adjusted for Receipt of Other Routine Tests

Event rate per 100 beneficiaries	Adjusted cascade-attributable event rate (95%CI)		
	**ECG**	**UA**	**TSH**
All events	6.18 (4.82, 7.54)	4.57 (3.38, 5.76)	0.28 (-0.66, 1.22)
Laboratory tests	0.68 (-0.20, 1.56)	1.19 (0.53, 1.85)	0.90 (0.38, 1.42)
Imaging	1.59 (1.04, 2.13)	0.94 (0.41, 1.48)	-0.18 (-0.39, 0.02)
Procedures	1.92 (1.42, 2.43)	0.83 (0.45, 1.21)	-0.09 (-0.44, 0.26)
Visits	1.25 (0.82, 1.67)	1.15 (0.55, 1.75)	-0.17 (-0.72, 0.38)
New diagnoses	0.47 (0.16, 0.78)	0.46 (0.14, 0.77)	0.17 (-0.06, 0.40)
Allowable charges related to cascade events in 90-day period excluding hospitalizations, $ mean (SD)[c]	9.59 (6.40, 12.78)	6.68 (4.24, 9.13)	-0.32 (-1.54, 0.89)

Results adjusted for patient age, sex, race, Medicaid, disability, Elixhauser condition count, residential setting, region, area-level income, ACO participation, specialty of clinician performing visit, visit co-billed status, HRR random effects (as in main analyses), as well as patient receipt of two other common routine tests: complete blood count (CPT codes: 85025, 85027) and metabolic panel (CPT codes: 80048, 80053, 80076).

eTable 11. Index Test and Cascade-Attributable Event Rates Following Routine Testing During Annual Wellness Visits, Sensitivity Analysis Using Test-Specific Cohorts

	**ECG (N = 174,171)**	**UA (N = 463,273)**	**TSH (N = 292,773)**
Index tests, (%)	18,953 (10.9%)	84,322 (18.2%)	44,472 (15.2%)
**Cascade-attributable event rate per 100 beneficiaries (95% CI)**			
All events	3.41 (2.64, 4.18)	2.63 (2.21, 3.04)	-0.53 (-0.84, -0.22)
Laboratory tests	0.36 (-0.15, 0.87)	1.24 (0.98, 1.51)	-0.36 (-0.49, -0.22)
Imaging	1.10 (0.79, 1.41)	0.47 (0.28, 0.66)	-0.02 (-0.10, 0.06)
Procedures	1.00 (0.70, 1.30)	0.33 (0.21, 0.45)	-0.15 (-0.27, -0.02)
Visits	0.85 (0.60, 1.10)	0.41 (0.22, 0.59)	0.19 (-0.03, 0.40)
New diagnoses	0.21 (0.03, 0.38)	0.08 (-0.01, 0.17)	-0.01 (-0.06, 0.04)

Results adjusted for patient age, sex, race, Medicaid, disability, Elixhauser condition count, residential setting, region, area-level income, ACO participation, specialty of clinician performing visit, visit co-billed status, and HRR random effects (as in main analyses).

eReferences

1. Society of General Internal Medicine. Choosing Wisely: General Health Checks for Asymptomatic Adults. https://www.choosingwisely.org/clinician-lists/society-general-internal-medicine-general-health-checks-for-asymptomatic-adults/. Published 2013. Accessed July 11, 2019.
2. Society of General Internal Medicine. Health Checkups. https://www.choosingwisely.org/patient-resources/health-checkups/. Published 2013. Accessed July 11, 2019.
